# Combined transorbital neuroendoscopic and endoscopic endonasal resection of orbital hemangioma

**DOI:** 10.3171/2025.1.FOCVID24197

**Published:** 2025-04-01

**Authors:** Christina Jackson, David Fernandes Cabral, S. Tonya Stefko, Eric W. Wang, Georgios A. Zenonos, Carl H. Snyderman, Paul A. Gardner

**Affiliations:** Departments of 1 Neurological Surgery,; 2 Ophthalmology, University of Pittsburgh Medical Center, Pittsburgh, Pennsylvania and; 3 Otolaryngology, University of Pittsburgh Medical Center, Pittsburgh, Pennsylvania

**Keywords:** transorbital neuroendoscopy, endoscopic endonasal approach, transorbital approach, orbital hemangioma

## Abstract

Orbital cavernous hemangiomas are the most common benign primary neoplasm of the orbit in adults and can lead to proptosis and optic neuropathy. Resection is the primary treatment for symptomatic cases. Choice of surgical approach depends on the lesion’s size, location, and relation to intraorbital structures. The authors present the case of a 40-year-old male with a large symptomatic orbital hemangioma resected through a combined endoscopic transorbital and endonasal approach. This case highlights the advantages of the combined approach, enhancing visibility and minimizing disruption of orbital structures, allowing for en bloc resection of large orbital hemangiomas.

The video can be found here: https://stream.cadmore.media/r10.3171/2025.1.FOCVID24197

## Transcript

We present a case of a large orbital cavernous hemangioma resected through a combined transorbital neuroendoscopic and endoscopic endonasal approach.^1^

### 0:31 Case Presentation.

The patient is a 40-year-old male who presented with right proptosis and vision loss for about 5 months. He also endorsed double vision with right lateral gaze.

### 0:41 Physical Examination.

Neuro-ophthalmology evaluation demonstrated right optic neuropathy with evidence of optic nerve edema on optical coherence tomography as well as inferior visual field loss of the right eye.

### 0:54 Imaging Findings.

MRI workup revealed a well-defined T2 hyperintense medial intraconal lesion. On early post-gadolinium sequences, the lesion initially demonstrated patchy enhancement. On subsequent delayed postcontrast sequences, it demonstrated progressive filling to a homogeneously enhancing lesion. This progressive filling is typically considered a pathognomonic radiographic sign of cavernous hemangiomas.^2,3^

### 1:18 Approach Consideration.

Various approaches have been described for intraorbital hemangiomas, and the choice of the approach depends on the lesion’s size, location, and relation to intraorbital structures.^4,5^ This lesion in particular is located in the medial inferior quadrant of the orbit. Given the vascular nature of these lesions, en bloc resection is typically preferred. Given the large size of this tumor and an already compromised right optic nerve, we wanted to minimize retraction and manipulation of the intraorbital contents. A natural dissection corridor in this case is between the medial and inferior rectus muscles coming medially. However, given the anterior extent of the tumor and to protect the rectus muscles, a transcaruncular endoscopic approach was also done to isolate the muscles, localize the tumor, and dissect the anterior aspect of the tumor. The medial orbitotomy then allows for orbital decompression and delivery of the tumor away from the critical centrally located optic nerve without retraction.^1^

### 2:18 Medial Orbitotomy.

We first began with a unilateral anterior and posterior ethmoidectomy and medial orbitotomy. The lamina papyracea on the right side was exposed to the orbital apex, identifying periorbita.

### 2:33 Transcaruncular Approach.

A transcaruncular approach was then performed to identify the medial and inferior rectus muscles. They were isolated with vessel loops to allow for mobilization. Once the intraconal corridor is developed, the anterior and lateral portions of the tumor were identified and dissected free from the intraorbital contents.

### 3:07 Opening Periorbita.

We then turned our attention to the endoscopic endonasal exposure. Using the vessel loops, we were able to identify the corridor between the medial and inferior rectus muscles. The periorbita was opened sharply along this corridor.

### 3:20 Tumor Dissection.

The extraconal fat is reduced using endoscopic bipolars. The medial and inferior rectus muscles can be gently retracted through the transcaruncular approach to widen the corridor. This also allows them to be less compressible and easier to dissect between. Using bimanual techniques, the tumor is then sharply dissected using endoscopic scissors. The tumor capsule was then bipolared and mobilized.

### 3:44 Tumor Delivery.

Working systematically circumferentially around the tumor, the remaining soft tissue tumor attachments are then incised, and the tumor is removed and delivered en bloc through the endonasal corridor.

### 4:09 Reconstruction.

Once the tumor is removed, hemostasis is obtained. The extraconal fat is placed back over the muscles to prevent scarring, and a free mucosal graft is placed over the area for reconstruction. The transcaruncular approach requires no closure.

### 4:30 Postoperative Course.

Postoperative MRI demonstrated complete tumor resection without evidence of residual disease. The patient had improvement in proptosis, and ophthalmology evaluation demonstrated resolution of right optic nerve swelling and neuropathy. The patient had an excellent cosmetic result with no evidence of extraocular movement dysfunction.

### 4:50 Conclusions.

Our case highlights the advantages of a combined transorbital neuroendoscopic and endoscopic endonasal approach for resection of intraorbital lesions in the inferior medial quadrant. The endoscopic endonasal approach minimizes external scarring with improved cosmesis. It also allows for enhanced visibility and minimal disruption of intraconal contents, while the transcaruncular approach allows for early identification and isolation of surrounding rectus muscles, which widens the natural corridor for a safe and effective en bloc resection.

## Disclosures

Dr. Jackson reported personal fees from Stryker as a consultant outside the submitted work. Dr. Snyderman reported ownership of stock options from SPIWay, LLC, outside the submitted work. Dr. Gardner reported personal fees from Peter Lazic US Inc., Sutter Medizintechnik, Mizuho, and Stryker Instruments, outside the submitted work and ownership of stock options from SPIWay, LLC.

## Author Contributions

Primary surgeon: Gardner. Assistant surgeon: Jackson, Fernandes Cabral, Stefko, Wang, Snyderman. Editing and drafting the video and abstract: Jackson, Fernandes Cabral. Critically revising the work: Gardner, Jackson, Fernandes Cabral, Wang, Zenonos, Snyderman. Reviewed submitted version of the work: all authors. Approved the final version of the work on behalf of all authors: Gardner. Supervision: Zenonos.

## Supplemental Information

### Patient Informed Consent

The necessary patient informed consent was obtained in this study.
